# Characterization of the Nano-Rod Arrays of Pyrite Thin Films Prepared by Aqueous Chemical Growth and a Subsequent Sulfurization

**DOI:** 10.3390/ma15196946

**Published:** 2022-10-06

**Authors:** Mohammad Talaeizadeh, Seyyed Ali Seyyed Ebrahimi, Payam Khosravi, Bejan Hamawandi

**Affiliations:** 1 Advanced Magnetic Materials Research Center, School of Metallurgy and Materials, College of Engineering, University of Tehran, Tehran 1417614411, Iran; 2 Department of Materials Engineering, Isfahan University of Technology, Isfahan 8415683111, Iran; 3 Department of Applied Physics, KTH Royal Institute of Technology, SE-106 91 Stockholm, Sweden

**Keywords:** FeS_2_, aqueous chemical growth, pyrite, nano-rod array, thin films

## Abstract

Pyrite is an earth-abundant and low-cost material with a specific collection of properties including a low band gap and high absorption coefficient of solar light. These properties make pyrite a good choice in a wide variety of applications such as catalysts, batteries, and photovoltaic devices. A thin film composed of vertically aligned pyrite nano-rods was processed via a hydration-condensation method followed by subsequent aging and sulfurization. In this process, no ionic salt was used which resulted in a lower cost process with a lower level of impurities. Field emission scanning electron microscopy, X-ray diffraction, and Raman spectroscopy analyses were used to characterize the thin films in different steps of the process. The major impurity of the final thin films was the marcasite phase according to the Raman analysis which could be minimized by lowering sulfurizing time to about 60 min. In addition, after structural, electrical, and optical characterization of thin films, these layers’ performances in a photovoltaic device were also examined. After deposition of a thin aluminum layer, Schottky-type solar cells of pyrite formed which were then illuminated to measure their current-voltage characteristics. The results show that a combination of low-cost materials and a low-cost preparation method is applicable for building future solar cells.

## 1. Introduction

From industrial and economical points of view, metal sulfides have gained increasing importance due to their remarkable physical, electrical, and chemical characteristics. Pyrite (FeS_2_), a polymorph of iron sulfide, is a good candidate for use in solar cells since it has excellent solid-state characteristics [1]. The crystal structure of the mineral pyrite (β-FeS_2_) is face-centered cubic and pyrite belongs to the $\;Pa\bar{3}$ space group [1]. The unit cell of pyrite contains four FeS_2_ formula units [2].The pyrite unit cell can be defined completely by $a_{0}$ (the lattice constant) and *u* (the S coefficient) that indicate the position of the Pyrite atom in the unit cell. In 1914, Bragg [3] introduced the complete crystal structure of a pyrite unit cell for the first time, and now the well-accepted values of $a_{0}$ and u are 5.416Å and 0.385Å, respectively [4,5]. Natural pyrites can be n-type or p-type (usually with arsenic), depending on the impurity type that they contain. The measured conductivity of natural pyrite is in the range of $0.02-562\;{\left(\Omega cm\right)}^{-1}$ [6] and its measured band gap is about 0.9 eV [2].

Nanocrystals of pyrite were successfully prepared two decades ago for the first time [7]. Recently, large [8] and small [9] clusters of pyrite nanocrystals have been reported.

Nevertheless, so far, no efficient pyrite-based solar cells have been reported. It has been claimed that the main drawback is pyrite’s low illuminated open-circuit voltage (lower than 0.2 V) which is the consequence of its inherent low unilluminated short-circuit current, due to the existence of impurities and the high density of surface states of pyrite [10].

So far, many methods have been used to synthesize pyrite [11,12,13,14,15,16,17]. Some of the primary methods used to prepare single crystals of pyrite are chemical vapor transport (CVT) and high-temperature solution using tellurium as the solvent [18].

According to Ostwald’s law, the production of pyrite by sulfurization of ferrous compounds starts from the production of iron sulfide (FeS), and as more sulfur diffuses the structure, iron disulfide (FeS_2_) gradually forms. Since the diffusion coefficient of sulfur atoms in iron is high ($D\;\approx \;5\times 10^{-7}\;cm^{2}/s$), FeS formation would occur immediately. However, the diffusion coefficient of sulfur in FeS is much lower than this, and thus, the formation of FeS_2_ takes a longer time [18].

To date, there have been many methods reported for producing pyrite thin films, including iron sulfurization, metal-organic chemical vapor deposition, and sintering of nanocrystals that have been prepared using the solvothermal method [19,20,21].

A report conducted by Wan et al. [22], in 2003, showed that the crystal structure, crystallization, stoichiometry, and more importantly, the preferential orientation of the crystal grains could affect the electrical and optical properties of pyrite thin films. Moreover, they demonstrated that it was the crystal grain size that specified the electrical properties, not the particle size. A year later, Ouertani et al. [23] successfully prepared a pyrite thin film by sulfurization of a layer of amorphous iron oxide which had been deposited previously by spray pyrolysis.

Ennaoui and Tributsch [24], in 1984, for the first time, showed the potential of crystalline pyrite to prepare a photoelectrochemical solar cell. Although the final cell they made could not reach over 1% conversion efficiency, they declared that the low raw material cost of pyrite and its inherent properties could make it a promising choice for use in the production of low-cost solar cells. They used pyrite as a solar cell’s synthesizer about a decade later and made a p-i-n structure solar cell [18]. They sandwiched a very thin layer (10–20 nm thick) of pyrite between a p-type and an n-type semiconductor. The analysis showed that pyrite had a spectacular electron conduction feature through interfaces.

In 2009, a hybrid solar cell was made by using pyrite nanocrystals as small as 10 nm, which had been prepared by a chemical solution method [9]. The conversion efficiency of this solar cell was 0.16%. Recently, cubic nanocrystals of pyrite, with 60–200 nm diameters, were used to prepare a Schottky-type photovoltaic solar cell with aluminum as the metallic contact [25]. The current-voltage analysis showed no Schottky barrier. Steinhagen et al. [26] used pyrite nanocrystals to make photovoltaic cells with four structures: Schottky, layered, depleted heterojunction, and hybrid. None of the structures showed a photovoltaic response.

Because of better catalytic activity, it has been claimed that the pyrite nano-rods have a higher short-circuit current in dye-synthesized solar cells as compared with pyrite layers [12].

A chemical procedure reported by Vayssieres et al. [27] has been recently used to grow nano-rod arrays of iron hematite ($\alpha -Fe_{2}O_{3}$) on any surface. The strategy involves controlling the thermodynamics and kinetics of interfaces during nucleation and growth which leads to good control of the size, shape, and structure of the final layer [28]. The process is called aqueous chemical growth (ACG) [29]. Precise control of the rate of heterogeneous nucleation and growth can result in one-dimensional growth and rod-like morphologies. Huang et al. [12] used the same method with a further sulfurizing step to obtain pyrite nano-rod arrays.

In this paper, pyrite nano-rod arrays were prepared by developing a novel ACG process followed by calcination and sulfurization steps. The structure of the resulting layers, after each step, was characterized and the structural, electrical, and optical properties of the final layers were investigated to apply this semiconductor in photovoltaic devices. In addition, the combination of pyrite nano-rods and the Schottky-type solar cell is a novel way to prepare a solar cell. It is noteworthy to mention that, in this research, for the first time, no ionic salt was used for the ACG process. The ACG process electrolyte pH was merely controlled by HCl. The results pave the way for future low-cost solar cells.

## 2. Experimental Procedures

The precursors $FeCl_{3}\cdot 6H_{2}O$, pyrite, and *HCl* were purchased from Merck and used as received. Fluorine-doped tin oxide (FTO)-coated glass with 8 ohms electrical resistance was purchased from Solaronix. To prepare β−FeOOH, the ACG method was established. The process started with 0.1 M iron chloride via mixing 5.4 g of $FeCl_{3}\cdot 6H_{2}O$ in 200 mL distilled water, followed by a pH reduction to 1.5 by adding *HCl*.

A reflux system was prepared to run ACG at 80 ℃. FTO- and non-coated glass were used inside this system as two different substrates. In all samples, an orange layer was formed on both sides of the substrates, and an orange-colored sediment was deposited in the flask simultaneously. The substrates were gently removed from the flask and were quickly washed with water. Meanwhile, the sediments were dried in an oven at 50 ℃ to obtain powder samples.

A muffle furnace was used for the calcination of the layered and powdered samples. Samples were tempered for 5 h at 400 ℃, and then they were cooled down to room temperature inside the furnace.

**Figure 1:**
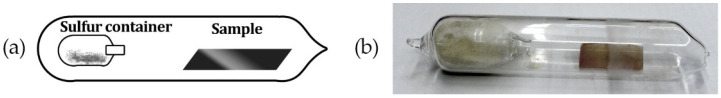
(**a**) Schematic; (**b**) real representation of quartz-sealed tubes for sulfurization process.

Then, the tempered samples were placed in a sealed quartz capsule with 1 g sulfur and were evacuated to 0.1 Torr, as depicted in Figure 1. Afterward, these capsules were placed in the furnace at 450 ℃ for different times, and they were cooled to room temperature inside the furnace. Then, the samples were removed from capsules. Table 1 summerizes sample coding system used in this research. The initial letter of the sample codes shows the sample type which means that glass, FTO-coated glass substrates, and powder sample codes start with G, F, and P, respectively. The number after this letter shows the process which was applied to the sample of that type. The second number shows the process variable (usually the processing time). G2 is the G17 sample after calcination. For more information about the process, please check the Supplementary Materials section.

The structural, electrical, and optical properties of samples from different steps of the preparation were examined. Samples G12, G14, G17, G3, and F31 were analyzed using a field emission scanning electron microscope (FESEM). The electrical resistance of G3 was measured using a four-probe surface resistance scanner. X-ray diffraction analyses were carried out for P1, P2, and P3 powders and also for Samples F1, F2, F36, F31, F3.30, F3.10, G1, G2, and G3. The X-ray spectrum was emitted from $Cu-K_{\alpha }$ with 40 kV voltage and 30 mA current. Samples G3, F36, F31, F3.30, and F3.10 were used for Raman analysis with a 10 MW laser with 785 nm wavelength to analyze the thin films. A UV-Vis analysis was also carried out for G3 with a light wavelength range of 190–1100 nm.

Furthermore, the performance of the prepared pyrite layers in a photovoltaic device was also examined. To examine the photovoltaic response, a 100 nm thick aluminum layer was deposited on the samples with FTO-coated glass substrate by using thermal evaporation. The current-voltage tests were carried out for final Schottky cells under airmass 1.5 with a $100\;\mathrm{mW}/{\mathrm{cm}}^{2}$ illumination of a solar simulator.

## 3. Results and Discussion

**Figure 2:**
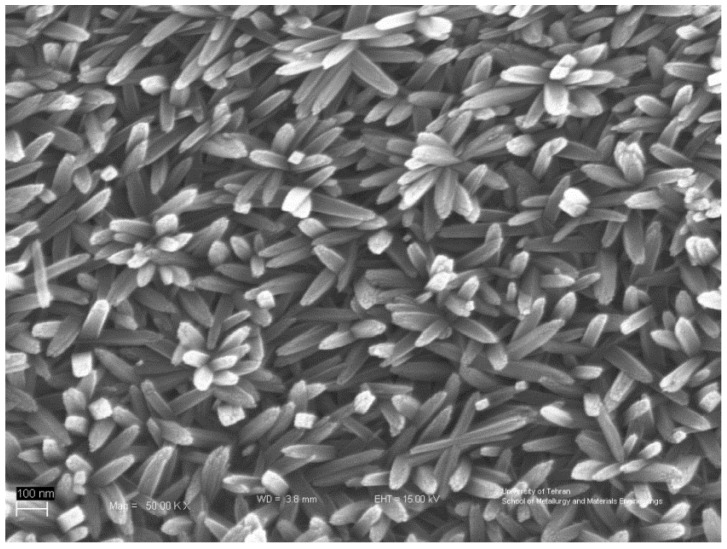
FESEM image of sample FeOOH thin film (Sample G17). A large number of flower-like nano-rods are formed on the substrate.

After the ACG process, an orange semi-transparent layer was formed on the substrates. The FESEM images of Sample G17 show a large number of flower-like nano-rods which are formed on the substrate, as shown in Figure 2. The mean diameter and length of the rods were 36 nm and 235 nm, respectively, measured from the FESEM images, and therefore, the mean aspect ratio of the nano-rods was 6.5.

**Figure 3:**
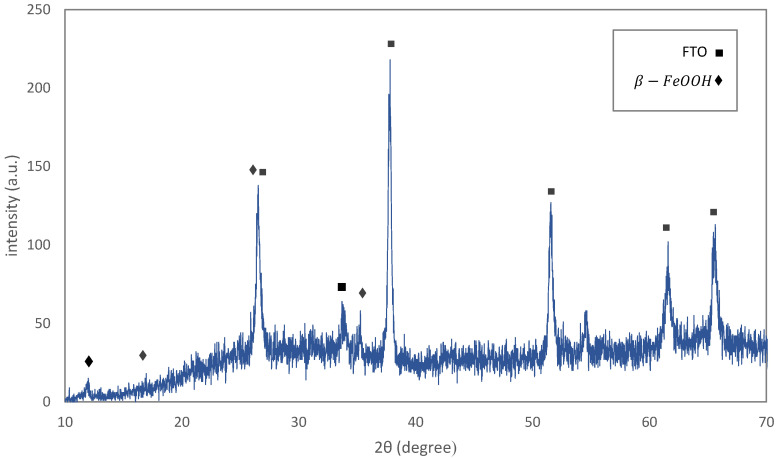
The XRD pattern of the sample β-FeOOH as grown on fluorine-doped tin oxide (FTO) substrate.

**Figure 4:**
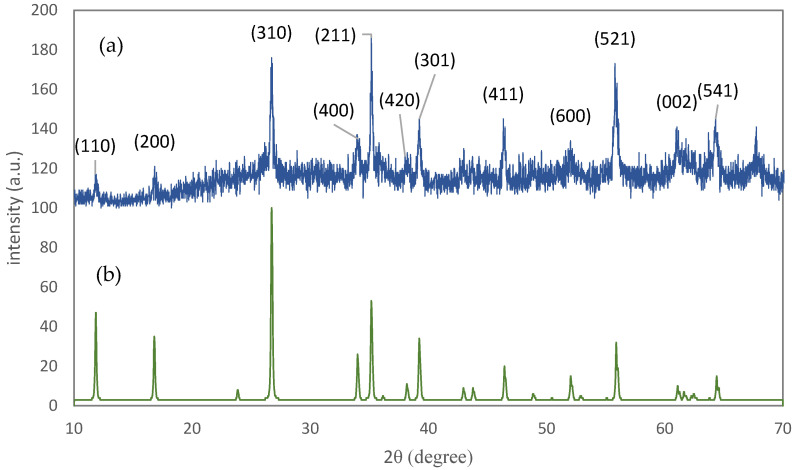
(**a**) The XRD pattern of the powder sample of FeOOH; (**b**) the simulated reference pattern of akaganéite phase according to the JCPDS card number 34-1266.

**Figure 5:**
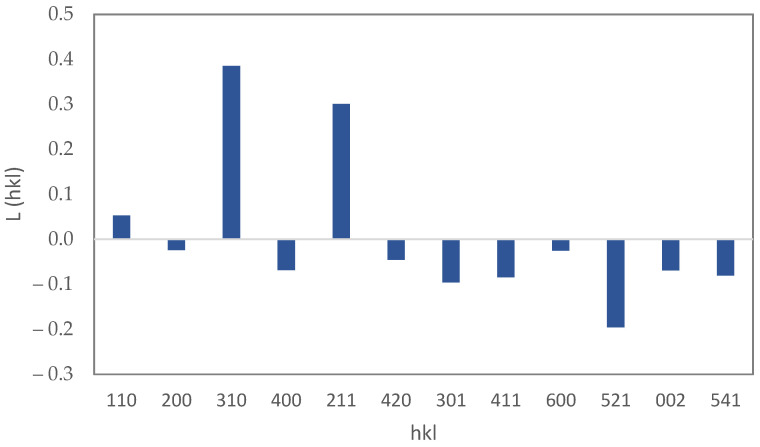
The Lotgering factor, L(hkl), for different crystallographic directions for the thin film sample of FeOOH.

The XRD pattern of Sample F1 is shown in Figure 3. The characteristic peaks of fluorine-doped tin oxide (FTO) and iron oxy-hydroxide (akaganéite) are visible in the pattern. This polymorph of FeOOH forms when a small amount of Cl is present in the crystal [30] which, in our solution, was supplied by HCl. The high intensity of FTO peaks may be due to the high thickness of this layer in contrast with the iron oxy-hydroxide layer. The XRD pattern of the powder sample, Sample P1, was used to examine the crystallite size and preferential orientation of the nano-rods (Figure 4). The crystallographic direction of each peak is labeled in Figure 4 according to the reference pattern. The average crystallite size, which can be estimated from the empirical Scherrer equation, is 37±3 nm. By compareing this average crystallite size with the particle size measured from microscopic images, it can be said that each nano-rod contains six crystallographic grains, on average. The Lotgering factor, L(hkl), was also used to measure the preferential orientation of the nano-rods [31]. The thin film’s orientation is totally parallel to the hkl orientation if L(hkl) equals one, and is random if L(hkl) equals zero. The calculated Lotgering factor was compared for the major crystallographic directions of Sample F1 in Figure 5 by using the XRD patterns of the powder sample (P1) and the thin film sample (F1). The L factors for the 310 and 211 directions are higher than the other directions. The 310 direction could be omitted because of its overlap with the FTO peak. A higher L factor in some directions means that the akaganéite nano-rods are preferentially oriented. However, in the literature, it has been reported that the orientation is parallel to the c axis [32,33]. This contradiction may be due to the lack of use of ionic salts in the ACG process. Ionic salts may adhere to some crystal facets and slow down their growth rate.

**Figure 6:**
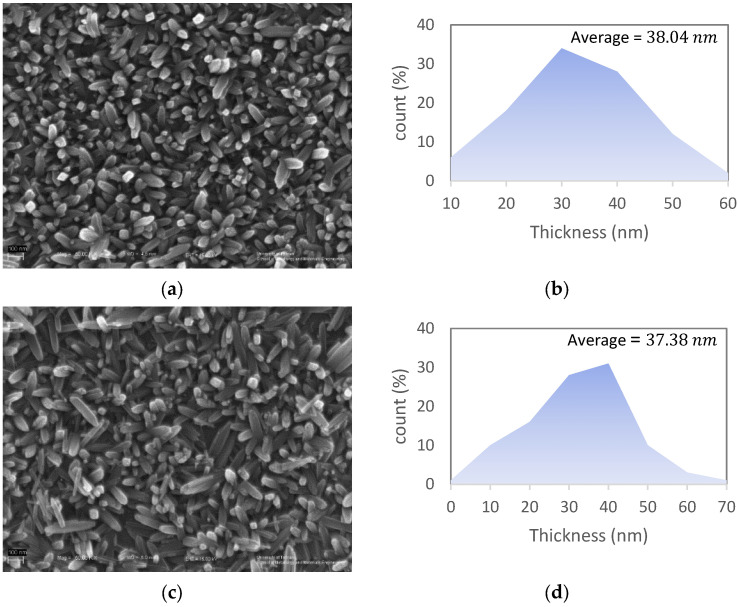

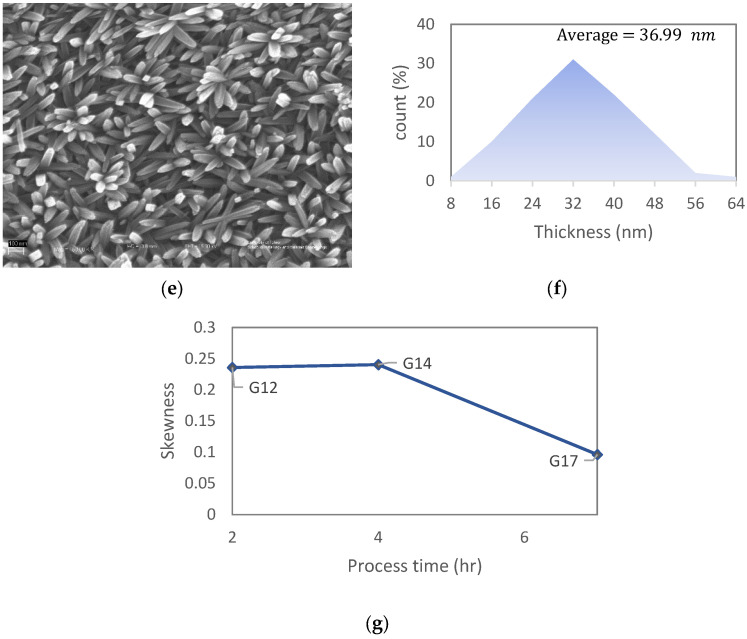
FESEM images of samples with: (**a**) 2 (Sample G12); (**c**) 4 (Sample G14); (**e**) 7 (Sample G17) hours of ACG process time along with digitally measured nano-rod thickness histograms (**b**,**d**,**f**), respectively; (**g**) calculated skewness factor of Samples G12, G14, and G17 nano-rod thickness histograms vs. process time.

The effect of the ACG process time on morphology was also studied. Samples with 2 (G12), 4 (G14), and 7 (G17) hours of process time are compared in Figure 6. It can be concluded that the ACG process time can affect the diameter distribution of the nano-rods, based on the demonstrated nano-rod thickness histograms in Figure 6b–f. The skewness factor was calculated for different samples, as shown in Figure 6g. The skewness factor is a measure of the asymmetry of a distribution. The sample with a 7 h ACG process time has a lower skewness factor than other samples, which means that the thickness distribution of the nano-rods in Sample G17 is more uniform.

**Figure 7:**
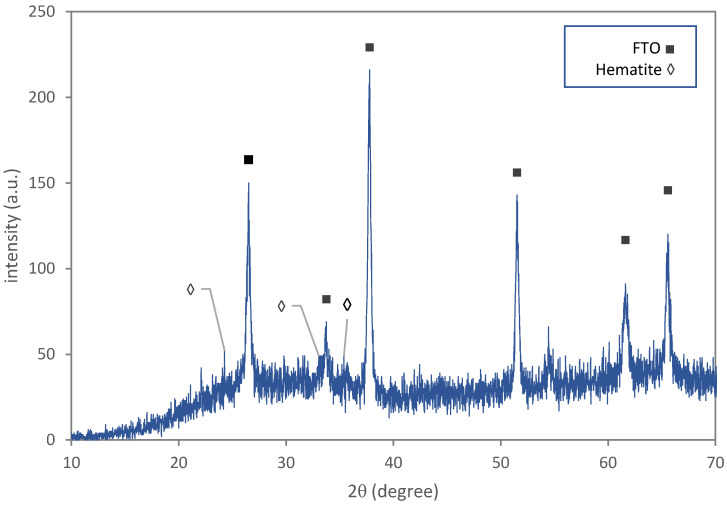
The XRD pattern of the thin film sample with FTO-coated glass substrate after calcination.

**Figure 8:**
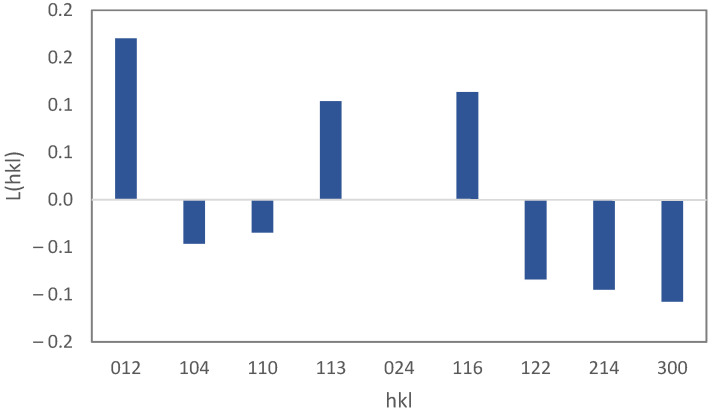
The Lotgering factor, L(hkl), for different crystallographic directions for the thin film sample after calcination.

The XRD pattern of the thin film with an FTO sublayer after calcination (F2) revealed two different phases: FTO and hematite ($\alpha -Fe_{2}O_{3}$), as shown in Figure 7. During calcination, water molecules abandon the akaganéite phase and the crystal structure transforms from tetragonal (I4/m) to trigonal (R$\bar{3}$c), and finally the hematite phase starts to form. The hematite crystallite size, according to the Scherrer equation, is 42±3 nm, which suggests a little grain growth during calcination. The calculated Lotgering factors for different crystal directions of the thin film sample (F2), according to the XRD pattern of the powder sample (P2), are shown in Figure 8. The preferential crystallographic direction of hematite nano-rods is [012].

**Figure 9:**
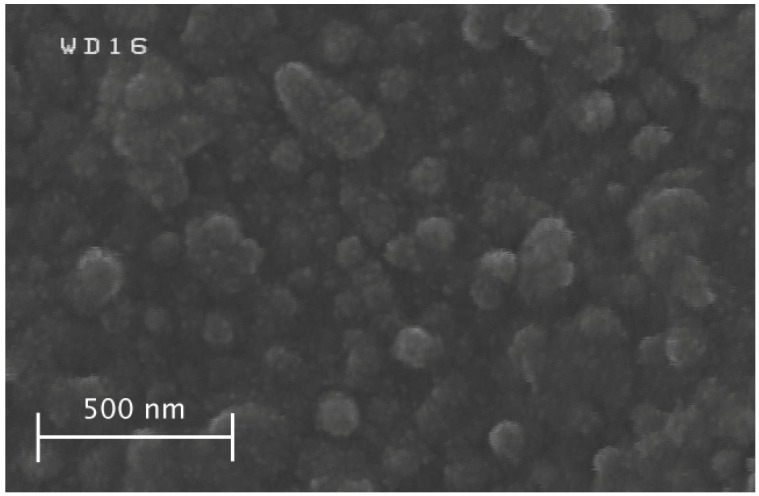
Top FESEM image of the thin film sample after sulfurization (Sample G3).

**Figure 10:**
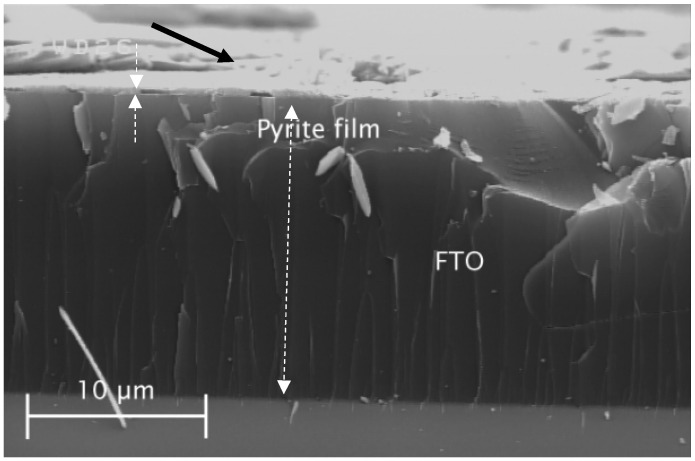
The SEM Image of the cross-section of pyrite thin film on FTO. The thickness of the two layers, FTO and pyrite, is compared by dashed arrows. The black arrow shows some impurities which are deposited on the surface of the thin film.

**Figure 11:**
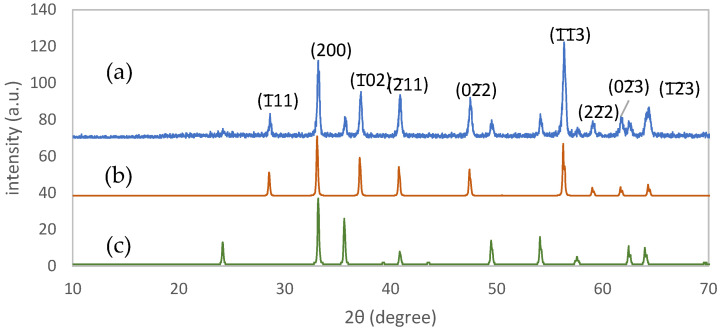
(**a**) The XRD pattern of pyrite powder sample with simulated patterns of reference cards of (**b**) pyrite (JCPDS 3-065-1211) and (**c**) hematite (JCPDS 33-0664).

The color of all the powder and thin film samples turned black after sulfurization. The FESEM image of Sample G3 from the top view of the thin film sample showed the nano-rods, as depicted in Figure 9. The diameter of the nano-rods was measured using an image software (ImageJ) and was 74 nm on average, which was two times larger than the thickness of akaganéite nano-rods. The thickness of this layer was between 400 and 700 nm according to its cross-section microscopic image shown in Figure 10. Moreover, some impurities were deposited on the thin film which could be the sulfur condensed residuals, shown by a black arrow in Figure 10. The XRD analysis of the powder sample (P3) showed an incomplete transformation of hematite to pyrite (FeS_2_) after sulfurization (Figure 11). The calculated crystallite size of pyrite crystals was 37±5 nm which was about 5 nm smaller than that of Sample P2 hematite crystallite size. Smaller pyrite crystals mean that not all of the hematite crystal was transformed to pyrite, and thus, the sulfurization time was insufficient. It has been reported that the main factor which affects the kinetics of the process is the diffusion rate of sulfur atoms into the hematite crystals and oxygen atoms out of it [18].

**Figure 12:**
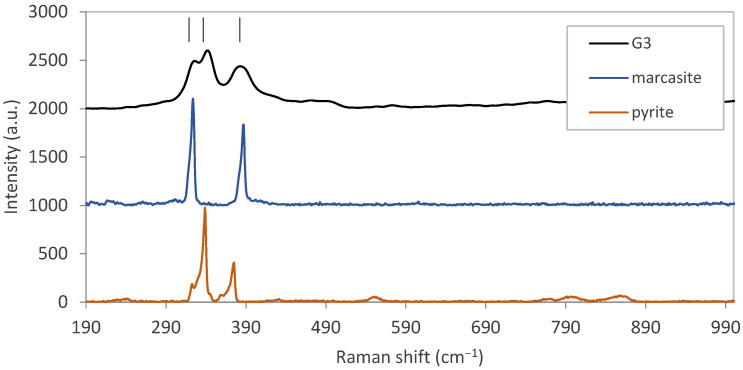
Raman shift for pyrite thin film on a glass substrate with the reference patterns of Marcasite and pyrite.

Marcasite is an iron sulfide phase with an orthorhombic crystal structure. A Raman spectroscopy analysis is the only way to distinguish between marcasite and pyrite phases, and it was used in this report to characterize the thin film Sample G3, as shown in Figure 12. It has been reported that the active Raman peaks of pyrite and marcasite are (353,387 cm^−1^) and (324,387 cm^−1^), respectively [34]. Accordingly, the first peak of the sample could be assigned to marcasite, the second one to pyrite, and the third one to both of them which was overlapped. Therefore, the sample layer consisted of both pyrite and marcasite phases. Pyrite was the dominant phase due to the relative Raman peak intensities. The formation of the marcasite phase in this process could be justified by the uncontrollable partial pressure of gaseous pyrite in the silicon tube which could result in the stabilization of two marcasite and pyrite phases alongside.

**Figure 13:**
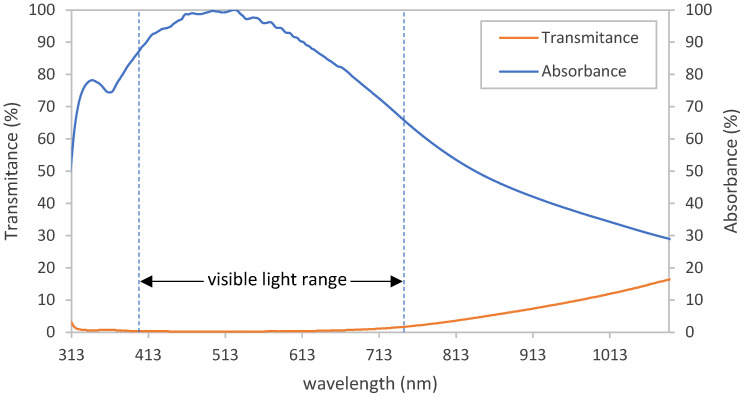
UV-Vis analysis of pyrite thin film on a glass substrate.

A UV-Vis analysis was carried out for Sample G3 to investigate the optical properties. The high absorption obtained in the UV to the visible range of the light wavelength emphasizes that pyrite is a good choice for the absorption of sunlight (Figure 13). In addition, no absorption peaks were observed in this range (313 to 1090 nm). In other words, the band gap of pyrite thin film is, at most, 1.127 eV.

**Figure 14:**
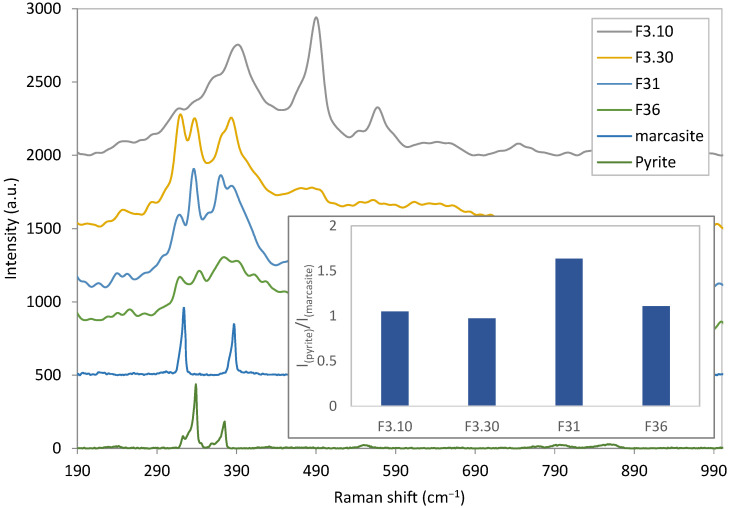
Raman shift for samples with different sulfurization times with reference Raman shifts of pyrite and marcasite. The inset is the chart of relative Raman first peak intensities of pyrite and marcasite for different samples.

The sulfurization process time varied and the Raman shifts of each sample were obtained (Figure 14). The samples all show Raman active peaks of pyrite and marcasite but there are also some unrecognized peaks visible in the curves. To estimate the relative contents of pyrite and marcasite, the ratios of the first active Raman peaks of pyrite and marcasite for different samples were calculated and compared in the inset in Figure 14. This ratio reveals that the sample with 1 h sulfurization time (F31) has the most pyrite to marcasite relative concentration.

**Figure 15:**
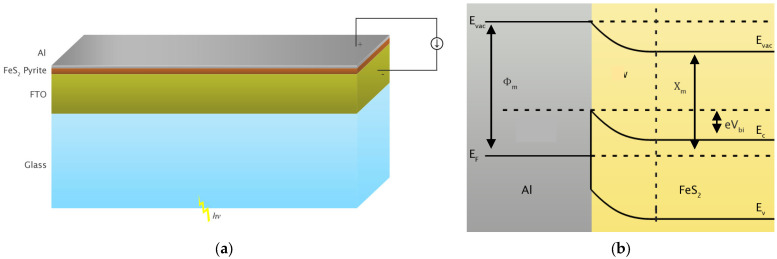
(**a**) A schematic representation of the final solar cell structure; (**b**) the estimated band diagram of the Schottky-type solar cell. Notice the bending of the valence (E_V_) and conduction (E_C_) bands of pyrite in contact with aluminum.

A schematic representation of the cross-section of the final solar cell is shown in Figure 15a. The structure consists of a thin film of pyrite which is sandwiched between an FTO-coated glass and a physical vapor-deposited aluminum layer. This is a Schottky-type solar cell in which a semiconductor (pyrite) is in contact with metal (aluminum). The aluminum and FTO layers also have the role of positive and negative electrodes, respectively.

The surface electrical resistance of pyrite, $R_{sh}$, was measured as $1.6\times 10^{5}\;\Omega /sq$, using a four-point probe ohmmeter. Furthermore, the calculated work function ($\phi_{m}$) of aluminum is 4.2 eV [35] and the electron affinity ($\chi_{sc}$) of pyrite is 3.28±0.14 eV [36]. The estimated band diagram of the contact of pyrite and aluminum is drawn by considering the above parameters in Figure 15b. The resulted band bending in the valence and conduction bands of pyrite in contact with the aluminum guide photoexcited electrons and holes toward the FTO and aluminum electrodes, respectively, before recombination occurs. This process completes the external circuit and produces electrical current. The maximum voltage that this cell can make is $eV_{bi}$, as shown in Figure 15b.

**Figure 16:**
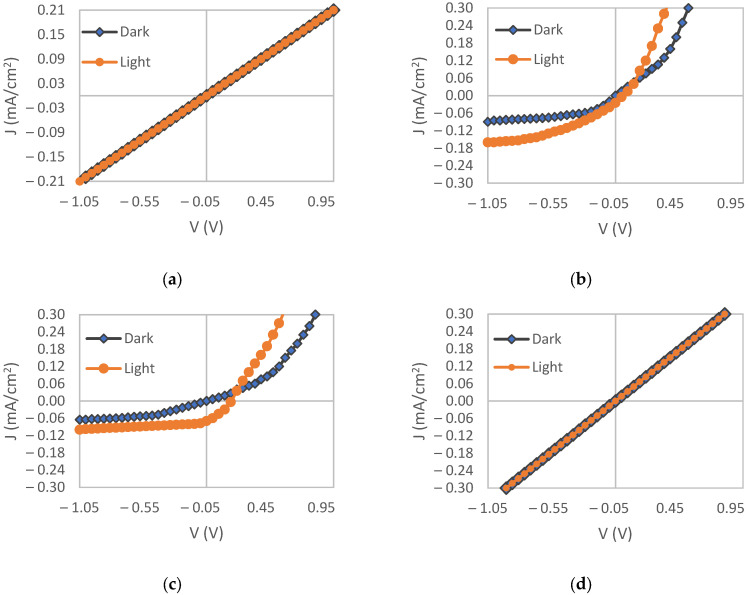
Current-voltage curves of: (**a**) F3.10; (**b**) F3.30; (**c**) F31; (**d**) F36, in dark and illuminated conditions.

The current-voltage behavior of each sample was measured in both dark and illuminated conditions. The resulting J-V curves are shown in Figure 16. Samples F3.10 (Figure 16a) and F36 (Figure 16d) did not show any photovoltaic response, and in particular, no Schottky contacts were detected. The electrical shunts might be responsible for this, as the film formation process is not fully controlled, and thus, the pyrite thin film may not cover all the substrate. Notice that the pyrite thin film thickness is as low as 500 nm and it is not possible to be increased by this type of ACG process.

Furthermore, unknown Raman peaks, as can be observed in Figure 14, can result from unknown chemical bonds that are formed during sulfurization. These conditions can cause new surface states or band defects which, in consequence, can lower the photoelectric response.

Samples F31 and F3.30 had photovoltaic responses according to Figure 16b,c. The calculated energy conversion efficiencies (η) of Samples F31 and F3.30 were 0.0044 and 0.00025%, respectively, which were lower than the η values reported by Lin [9] and Ennaoui [18] and were consistent with those reported by Steinhagen [26] and Bi [25]. 

Nevertheless, the η value of the sample with 1 h sulfurization time (F31) is higher than those of the other samples. This can be related to the higher pyrite to marcasite ratio of this sample. It can be stated that the main reason for the low open-circuit voltage of these solar cells is the presence of marcasite impurity, as reported previously [37]. Moreover, the formation of new surface states in pyrite can cause the recombination of excited electrons and holes before being collected in the external circuit [2,38].

## 4. Conclusions

In this study, an ionic salt-free ACG process followed by calcination and sulfurization was developed for preparing FeS_2_ thin films. The method is inexpensive and also enters lower impurity to the product. In the ACG step, the akaganéite nano-rod array thin film was formed. Akaganéite changed to hematite, and then pyrite, respectively, in the calcination and sulfurization steps. These transitions were detected by XRD; however, further studies by Raman spectroscopy proved the presence of the marcasite phase in the final layer. This phase is not desirable and is considered to be an impurity because it reduces the photovoltaic response of the layer. The pyritization time can affect the pyrite to marcasite ratio, which can be minimized for 60 min; therefore, the photovoltaic response of the Schottky-type solar cells made from pyrite thin film was improved by optimizing sulfurization times. The low-cost material and preparation method is a suitable combination for future solar cells. However, more investigations on the detailed photovoltaic mechanisms and characterizations are suggested before using these devices in real applications.

## Figures and Tables

**Table 1:** Sample coding system.

Sample Type	After the ACG Process (Process Time)	After Calcination Process	After Pyriteization Process (Process Time)
Glass substrate	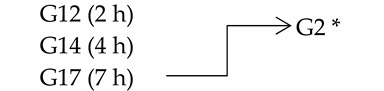		G3
FTO coated glass substrate	F1	F2	F36 (6 h) F31 (1 h) F3.30 (30 min) F3.10 (10 min)
Powder	P1	P2	P3 (6 h)

* Sample G2 is Sample G17 after calcination.

## Supplementary Materials

The following supporting information can be downloaded at: www.mdpi.com/article/10.3390/ma15196946/s1.

## References

[1] Barnard A.S., Russo S.P. (2007). Shape and Thermodynamic Stability of Pyrite FeS2 Nanocrystals and Nanorods. J. Phys. Chem. C.

[2] Murphy R., Strongin D.R. (2009). Surface Reactivity of Pyrite and Related Sulfides. Surf. Sci. Rep..

[3] Bragg W.H. (1914). The X-ray Spectra Given by Crystals of Sulphur and Quartz. Proc. R. Soc. London. Ser. A.

[4] Kleppe A.K., Jephcoat A.P. (2004). High-pressure Raman Spectroscopic Studies of FeS2 pyrite. Mineral. Mag..

[5] Stevens E.D., DeLucia M.L., Coppens P. (1980). Experimental Observation of The Effect of Crystal Field Splitting on The Electron Density Distribution of Iron pyrite. Inorg. Chem..

[6] Abraitis P.K., Pattrick R.A.D., Vaughan D.J. (2004). Variations in The Compositional, Textural and Electrical Properties of Natural Pyrite: A Review. Int. J. Miner. Process..

[7] Wilcoxon J.P., Newcomer P.P., Samara G.A. (1996). Strong Quantum Confinement Effects in Semiconductors: FeS2 Nanoclusters. Solid State Commun..

[8] Wadia C., Wu Y., Gul S., Volkman S.K., Guo J., Alivisatos A.P. (2009). Surfactant-Assisted Hydrothermal Synthesis of Single phase Pyrite FeS2 Nanocrystals. Chem. Mater..

[9] Lin Y.Y., Wang D.Y., Yen H.C., Chen H.L., Chen C.C., Chen C.M., Tang C.Y., Chen C.W. (2009). Extended Red Light Harvesting in a Poly(3-hexylthiophene)/iron Disulfide Nanocrystal Hybrid Solar Cell. Nanotechnology.

[10] Buker K., Alonso-Vante N., Tributsch H. (1992). Photovoltaic Output Limitation of n-FeS2 (Pyrite) Schottky Barriers: A Temperature-dependent Characterization. J. Appl. Phys..

[11] Sun K., Su Z., Yang J., Han Z., Liu F., Lai Y., Li J., Liu Y. (2013). Fabrication of Pyrite FeS2 Thin Films by Sulfurizing Oxide Precursor Films deposited via successive ionic layer adsorption and reaction method. Thin Solid Film..

[12] Huang Q.-H., Ling T., Qiao S.-Z., Du X.-W. (2013). Pyrite nanorod arrays as an efficient counter electrode for dye-sensitized solar cells. J. Mater. Chem. A.

[13] Yuan B., Luan W., Tu S. (2012). One-step synthesis of cubic FeS2 and flower-like FeSe2 particles by a solvothermal reduction process. Dalton Trans..

[14] Ma H., Zou Z.G., Wu Y., Long F., Yu H.J., Xie C.Y. (2011). Solvothermal Synthesis of Pyrite (FeS2). Adv. Mater. Res..

[15] Wang D.W., Wang Q.H., Wang T.M. (2010). Controlled growth of pyrite FeS2 crystallites by a facile surfactant-assisted solvothermal method. CrystEngComm.

[16] Roberts D.M., Russek S.E., Stoldt C.R. (2019). Synthetic iron pyrite across length scales: Interfacial defects and macroscopic properties. CrystEngComm.

[17] Botchway E.A., Ampong F.K., Nkrumah I., Boakye F.K., Nkum R.K. (2019). Growth of a pure and single phase iron sulfide (pyrite) thin film by electrochemical deposition for photovoltaic applications. Open J. Appl. Sci..

[18] Ennaoui A., Fiechter S., Pettenkofer C., Alonso-Vante N., Büker K., Bronold M., Höpfner C., Tributsch H. (1993). Iron Disulfide for Solar Energy Conversion. Sol. Energy Mater. Sol. Cells.

[19] Bausch S., Sailer B., Keppner H., Willeke G., Bucher E., Frommeyer G. (1990). Preparation of pyrite films by plasma-assisted sulfurization of thin iron films. Appl. Phys. Lett..

[20] Bouchard R.J. (1968). The preparation of single crystals of FeS2, CoS2, and NiS2 pyrites by chlorine transport. J. Cryst. Growth.

[21] Thomas B., Ellmer K., Bohne W., Röhrich J., Kunst M., Tributsch H. (1999). Photoeffects in cobalt doped pyrite (FeS2) films. Solid State Commun..

[22] Wan D., Wang Y., Wang B., Ma C., Sun H., Wei L. (2003). Effects of the crystal structure on electrical and optical properties of pyrite FeS2 films prepared by thermally sulfurizing iron films. J. Cryst. Growth.

[23] Ouertani B., Ouerfelli J., Saadoun M., Bessaïs B., Hajji M., Kanzari M., Ezzaouia H., Hamdadou N., Bernède J. (2005). Transformation of amorphous iron oxide films pre-deposited by spray pyrolysis into FeS2-pyrite films. Mater. Lett..

[24] Ennaoui A., Tributsch H. (1984). Iron sulphide solar cells. Sol. Cells.

[25] Bi Y., Yuan Y., Exstrom C.L., Darveau S.A., Huang J. (2011). Air Stable, Photosensitive, Phase Pure Iron Pyrite Nanocrystal Thin Films for Photovoltaic Application. Nano Lett..

[26] Steinhagen C., Harvey T.B., Stolle C.J., Harris J., Korgel B.A. (2012). Pyrite nanocrystal solar cells: Promising, or fool’s gold?. J. Phys. Chem. Lett..

[27] Vayssieres L., Beermann N., Lindquist S.-E., Hagfeldt A. (2001). Controlled Aqueous Chemical Growth of Oriented Three-Dimensional Crystalline Nanorod Arrays:  Application to Iron(III) Oxides. Chem. Mater..

[28] Vayssieres L., Rabenberg L., Manthiram A. (2002). Aqueous Chemical Route to Ferromagnetic 3-D Arrays of Iron Nanorods. Nano Lett..

[29] Vayssieres L. (2006). Advanced semiconductor nanostructures. Comptes Rendus Chim..

[30] Rémazeilles C., Refait P. (2007). On the formation of β-FeOOH (akaganéite) in chloride-containing environments. Corros. Sci..

[31] Lotgering F.K. (1959). Topotactical reactions with ferrimagnetic oxides having hexagonal crystal structures—I. J. Inorg. Nucl. Chem..

[32] Vayssieres L. (2004). On the design of advanced metal oxide nanomaterials. Int. J. Nanotechnol..

[33] Joly A.G., Xiong G., Wang C., McCready D.E., Beck K.M., Hess W.P. (2007). Synthesis and photoexcited charge carrier dynamics of Β-FeOOH nanorods. Appl. Phys. Lett..

[34] Mernagh T.P., Trudu A.G. (1993). A laser Raman microprobe study of some geologically important sulphide minerals. Chem. Geol..

[35] Grundmann M. (2006). The Physics of Semiconductors: An Introduction Including Devices and Nanophysics.

[36] Rienstra-Kiracofe J.C., Tschumper G.S., Schaefer H.F., Nandi S., Ellison G.B. (2002). Atomic and Molecular Electron Affinities:  Photoelectron Experiments and Theoretical Computations. Chem. Rev..

[37] Bedja I., Hagfeldt A. (2011). FeS2-Quantum-Dot Sensitized Metal Oxide Photoelectrodes: Photoelectrochemistry and Photoinduced Absorption Spectroscopy. Adv. OptoElectronics.

[38] Birkholz M., Fiechter S., Hartmann A., Tributsch H. (1991). Sulfur deficiency in iron pyrite (FeS_{2-x}) and its consequences for band-structure models. Phys. Rev. B.

