# Supplementary material for: Characterization of the Nano-Rod Arrays of Pyrite Thin Films Prepared by Aqueous Chemical Growth and a Subsequent Sulfurization
Source: Materials (Basel). 2022 Oct 6;15(19):6946. doi: 10.3390/ma15196946 (PMC9570830; doi:10.3390/ma15196946)
Supplement: Supplementary file 1 [file materials-15-06946-s001.zip › materials-1925916-supplementary.pdf]

materials

Supplementary Materials

Characterization of the Nano-Rod Arrays of Pyrite Thin Films Prepared by Aqueous Chemical Growth and Sulfurization

Mohammad Talaeizadeh [1], Seyyed Ali Seyyed Ebrahimi [1,*], Payam Khosravi [1,2] and Bejan Hamawandi [3,*]

1 Advanced Magnetic Materials Research Center, School of Metallurgy and Materials, College of Engineering, University of Tehran, Tehran 111554563, Iran
2 Department of Materials Engineering, Isfahan University of Technology, Isfahan 84156-83111, Iran
3 Department of Applied Physics, KTH Royal Institute of Technology, SE-106 91 Stockholm, Sweden
* Correspondence: saseyyed@ut.ac.ir (S.A.S.E.), bejan@kth.se (B.H.)

As mentioned in the experimental and procedure section, the precursors become the final product after 3 steps. The product of these steps encoded respectively by X1, X2, and X3 (where X stands for G, F, or P):

Raw materials $\rightarrow^{1}$ X1 $\rightarrow^{2}$ X2 $\rightarrow^{3}$ X3

In this section, we will introduce the reactions of each step.

The first step is ACG process which includes hydration and deposition. In this step $FeCl_3.6H_2O$, as precursor, participates in the following reaction in an aqueous solution:

$FeCl_3 + H_2O \rightarrow FeOOH\ (s) + 3HCl$ (1)

As a result β-FeOOH in form of Nano-rods is deposited. This product is called X1.

The second step is calcination. In this step X1 specimens were tempered and experience the following reaction:

$2FeOOH \rightarrow Fe_2O_3 + H_2O$ (2)

As the result, the Nano-rods' composition converts to α-Fe_2O_3 (Hematite). The product is called X2 at this step.

The final step is sulfurization. In this step X2 specimens were annealed in Sulfur containing atmosphere and the following reaction occurs:

$2Fe_2O_3 + 11S \rightarrow 4FeS_2\ (s) + 3SO_2\ (g)$ (3)

As the result, Nano-rods' chemical composition change into FeS_2. These specimens are called X3. FeS_2 crystal phase in X3 specimens is composed of a mix of pyrite and Marcasite.
